# NELL-1 Increased the Osteogenic Differentiation and mRNA Expression of Spheroids Composed of Stem Cells

**DOI:** 10.3390/medicina57060586

**Published:** 2021-06-08

**Authors:** Jong-Ho Lee, Young-Min Song, Sae-Kyung Min, Hyun-Jin Lee, Hye-Lim Lee, Min-Ji Kim, Yoon-Hee Park, Je-Uk Park, Jun-Beom Park

**Affiliations:** 1 Department of Oral and Maxillofacial Surgery, College of Medicine, The Catholic University of Korea, Seoul 06591, Korea; vonsman@naver.com; 2 Department of Periodontics, College of Medicine, The Catholic University of Korea, Seoul 06591, Korea; 22003897@cmcnu.or.kr (Y.-M.S.); msek1004@naver.com (S.-K.M.); 21700035@cmcnu.or.kr (H.-J.L.); 3 Department of Anatomy and Neurobiology, School of Medicine, University of California, Irvine, CA 92697, USA; hyelil1@uci.edu; 4 College of Dentistry, Chosun University, Gwangju 61452, Korea; mjkim1310@chosun.kr; 5 Ebiogen, #405, Sungsu A1 Center 48 Ttukseom-ro 17-ga-gil, Seongdong-gu, Seoul 04785, Korea; yhpark@e-biogen.com

**Keywords:** human NELL1 protein, osteogenesis, mesenchymal stem cells

## Abstract

*Background and objectives:* NELL-1 is a competent growth factor and it reported to target cells committed to the osteochondral lineage. The secreted, osteoinductive glycoproteins are reported to rheostatically control skeletal ossification. This study was performed to determine the effects of NELL-1 on spheroid morphology and cell viability and the promotion of osteogenic differentiation of stem cell spheroids. *Materials and Methods:* Cultures of stem cell spheroids of gingiva-derived stem cells were grown in the presence of NELL-1 at concentrations of 1, 10, 100, and 500 ng/mL. Evaluations of cell morphology were performed using a microscope, and cell viability was assessed using a two-color assay and Cell Counting Kit-8. Evaluation of the activity of alkaline phosphatase and calcium deposition assays involved anthraquinone dye assay to determine the level of osteogenic differentiation of cell spheroids treated with NELL-1. Real-time quantitative polymerase chain reaction (qPCR) was used to evaluate the expressions of *RUNX2*, *BSP*, *OCN*, *COL1A1*, and *β-actin* mRNAs. *Results:* The applied stem cells produced well-formed spheroids, and the addition of NELL-1 at tested concentrations did not show any apparent changes in spheroid shape. There were no significant changes in diameter with addition of NELL-1 at 0, 1, 10, 100, and 500 ng/mL concentrations. The quantitative cell viability results derived on Days 1, 3, and 7 did not show significant disparities among groups (*p* > 0.05). There was statistically higher alkaline phosphatase activity in the 10 ng/mL group compared with the unloaded control on Day 7 (*p* < 0.05). A significant increase in anthraquinone dye staining was observed with the addition of NELL-1, and the highest value was noted at 10 ng/mL (*p* < 0.05). qPCR results demonstrated that the mRNA expression levels of RUNX2 and BSP were significantly increased when NELL-1 was added to the culture. *Conclusions:* Based on these findings, we conclude that NELL-1 can be applied for increased osteogenic differentiation of stem cell spheroids.

## 1. Introduction

NELL-1 is a potent growth factor, reported to target cells committed to the osteochondral lineage [1]. NELL-1 is reported to be encoded by the NELL1 gene in humans, and human NELL1 has been mapped to chromosome 11 at 11p15.1–p15.2 [2]. NELL-1-deficient cranial neural crest cells exhibited a noticeable reduction in cellular proliferation along with a significant decrease in osteogenic differentiation [3]. NELL-1 can be applied in the healing of a bony defect through the enhancement of osteogenesis and repairment [4]. It has been shown that NELL-1-haploinsufficient mice exhibit normal skeletal development but experience age-related osteoporosis, characterized by increased bone fragility and a reduction in osteoblast to osteoclast ratio [5]. NELL-1 is reported to have additional effects on anti-adipogenic activities, and application of NELL-1 inhibited bone morphogenetic protein-9-induced adipogenesis [6]. NELL-1 is reported to induce odontoblast differentiation and pulp capping with enhanced formation of reparative dentin and reduced inflammatory cell responses, suggesting it as a positive regulator for pulp repair [7]. NELL-1 also is shown to exhibit anti-inflammatory effects and pro-chondrogenic dual functions [8].

In recent years, stem cell research has been of great interest, and the number of such studies has grown tremendously [9,10]. Stem cells have differentiation abilities including osteogenic differentiation [11]. In a previous report, infusion of mesenchymal stem cells improved bone performance as a result of stem cell differentiation into osteoblasts [12]. Mesenchymal stem cells can be applied due to paracrine effects, which is proven by using conditioned media, and this approach can be applied to regenerative medicine, especially in bones [13,14]. In a previous report, NELL-1 significantly induced mesenchymal stem cell migration [15]. Spheroid culture has the advantages of improvement in viability and proliferation and promotion of the stemness marker expression [16]. Spheroids are shown to facilitate cell-to-cell interaction and to produce an increased osteogenic potential [17]. The purpose of this study is to reveal the effects of NELL-1 on the morphology of spheroids, maintenance of cell viability, and enhancement of osteogenic differentiation using three-dimensional cultures of stem cells.

## 2. Materials and Methods

### 2.1. Fabrication of Stem Cell Spheroids

The Institutional Review Board approved the protocol of the present study after reviewing the document (No. KC19SISI0816 and KC20SISE0733; approved date: 20 November 2019). The study was conducted in accordance with the Declaration of Helsinki. Gingiva-derived mesenchymal stem cells (GMSCs) were obtained following previously reported methods [18]. Obtained gingival tissues were de-epithelialized, minced and digested with enzyme. The stem cells were loaded on a culture dish, and the culture media was changed every two to three days.

**Figure 1:**
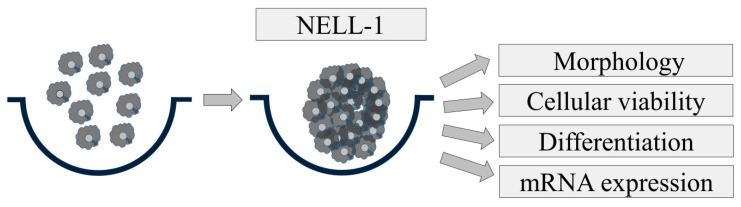
A general overview of the present study.

Figure 1 demonstrates a general view of the study design. Microwells (StemFIT 3D; MicroFIT, Seongnam, Korea) were used to formulate stem cell spheroids with a total of 1 × 10^6^ cells per well. Cell spheroids made of gingiva-derived stem cells were treated with NELL-1 (NELL-1810; 5487-NL, R&D Systems, Inc., Minneapolis, MN, USA) at the concentrations of 0, 1, 10, 100, and 500 ng/mL on the third day. Morphological evaluation of the spheroids was conducted on Days 1, 3, 5, and 7 using an inverted microscope, and the images were saved as JPEGs using the attached charge-coupled device camera. The intactness of the spheroids and the change in the diameter was evaluated regarding the morphological analyses. The diameter of the spheroids was calculated following the previous method [19]. Three spheroids were used for the measurements in each group at each time point.

### 2.2. Determination of Cell Viability

Two-color-based assays (Live/Dead Kit assay, Molecular Probes, Eugene, OR, USA) based on esterase activity and integrity of the plasma membrane were used for qualitative cell viability. The spheroids were incubated for 30 min at room temperature after adding 2 µL of 50 mM calcein acetoxymethyl ester working solution and 4 µL of 2 mM ethidium homodimer-1 [20]. Three spheroids were analyzed for each group at each time point. Spectrophotometric analyses using Cell Counting Kit-8 (Dojindo, Tokyo, Japan) were used for quantitative cell viability testing. The spheroids were cultured for 60 min at 37 °C after adding tetrazolium, and monosodium salt.

### 2.3. Activity of Alkaline Phosphatase and the Evaluation of Calcium Deposits

Cell spheroids were grown in a 37 °C humidified incubator containing 5% CO_2_ with the osteogenic media. The activity of alkaline phosphatase and anthraquinone dye staining were used to evaluate the osteogenic differentiation of stem cell spheroids on Days 7 and 14 [21]. Colorimetric analyses based on para-nitrophenylphosphate were used to evaluate the activity of alkaline phosphatase. The supernatant was mixed with a *p*-nitrophenylphosphate substrate and incubated at 25 °C for 60 min. The absorbance of the resultant *p*-nitrophenol was measured spectrophotometrically at 405 nm. 

Anthraquinone dye staining was used to assess calcium deposits. Stem cell spheroids were stained with anthraquinone dye for 30 min at room temperature after washing and fixation procedures. Bound dyes were quantified by spectrophotometric analysis after application of cetylpyridinium chloride.

### 2.4. Total RNA Extraction and Quantification of RUNX2, BSP, OCN, COL1A1 mRNA by Real-Time Quantitative Polymerase Chain Reaction (qPCR)

Quantity of RNA were performed on the extracted total RNA [22]. mRNA expression was detected by qPCR on Day 7. We used GenBank to design the sense and antisense primers for PCR. The primer sequences were as follows: *RUNX2* (accession No.: NM_001015051.3; forward: 5′-CAGTTCCCAAGCATTTCATCC-3′, reverse: 5′-AGGTGGCTGGATAGTGCATT-3′), *BSP* (accession No.: NM_004967.4; forward: 5′-CCTCTCCAAATGGTGGGTTT-3′, reverse: 5′-ATTCAACGGTGGTGGTTTTC-3′), *OCN* (accession No.: NM_199173.6; forward 5′-GGTGCAGAGTCCAGCAAAGG-3′, reverse: 5′-GCGCCTGGGTCTCTTCACTA-3′), COL1A1 (accession No.: NM_000088.4; forward: 5′-TACCCCACTCAGCCCAGTGT-3′, reverse: 5′-CCGAACCAGACATGCCTCTT-3′), and *β-actin* (accession. No.: NM 001101: forward: 5′-AATGCTTCTAGGCGGACTATGA-3′, reverse: 5′-TTTCTGCGCAAGTTAGGTTTT-3′) [23]. Gene expression of each mRNA was normalized to the endogenous reference gene β-actin as an internal control.

### 2.5. Statistical Analysis

We presented the data as mean ± standard deviation of the results. Tests of normality and equal variances were executed. The differences between the groups were analyzed using one-way analysis of variance with Tukey’s post hoc test with SPSS 12 for Windows (SPSS Inc., Chicago, IL, USA; *p* < 0.05). Three experimental replicates were evaluated for each analysis.

## 3. Results

### 3.1. Evaluation of Stem Cell Morphology and Determination of Cell Viability

**Figure 2:**
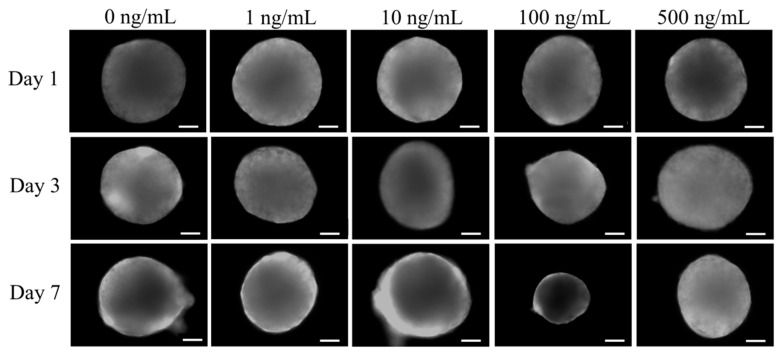
The morphology of the spheroids. The scale bar represents 200 μm (original magnification ×100).

The shapes of cells cultured in growth media on Days 1, 3, and 7 are shown in Figure 2. Addition of NELL-1 at 0, 1, 10, 100, and 500 ng/mL concentrations did not exhibit notable changes in spheroid shape.

**Figure 3:**
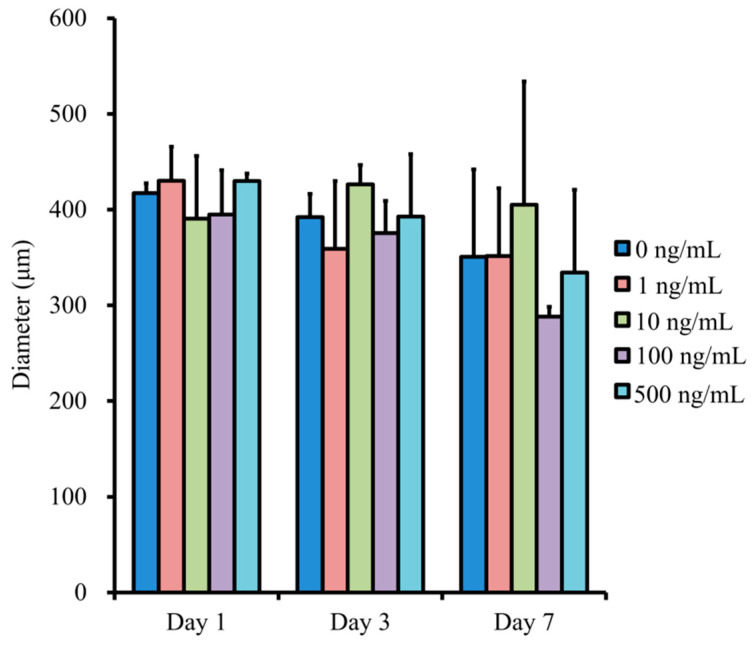
Evaluation of the diameter of the spheroids.

The changes in diameter of the spheroids are shown in Figure 3. There were no statistical differences with the addition of NELL-1 at 0, 1, 10, 100, and 500 ng/mL concentrations. The longer incubation did not produce statistical differences in diameter (*p* > 0.05).

**Figure 4:**
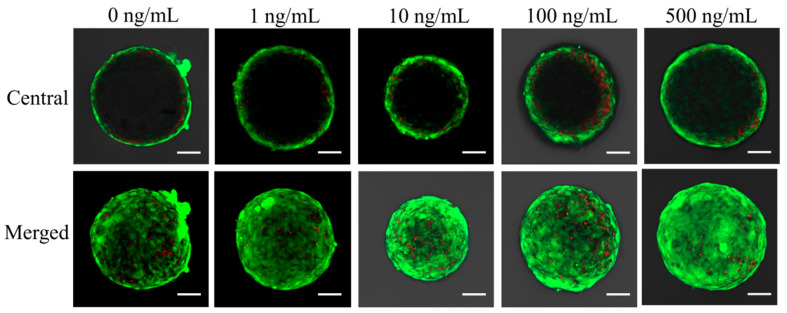
Results of central and merged images of live and dead cells of stem cell spheroids on Day 1. Intracellular esterase produces green fluorescence in intact cells and ethidium homodimer demonstrates red fluorescence in damaged cells. The scale bar indicates 100 μm (original magnification ×200).

**Figure 5:**
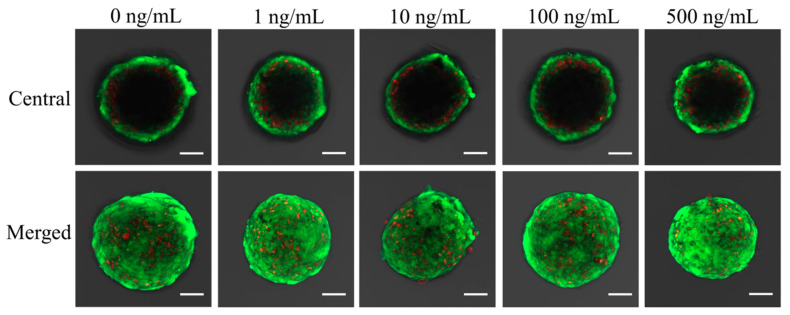
Results of central and merged images on Day 3. The scale bar represents 100 μm.

**Figure 6:**
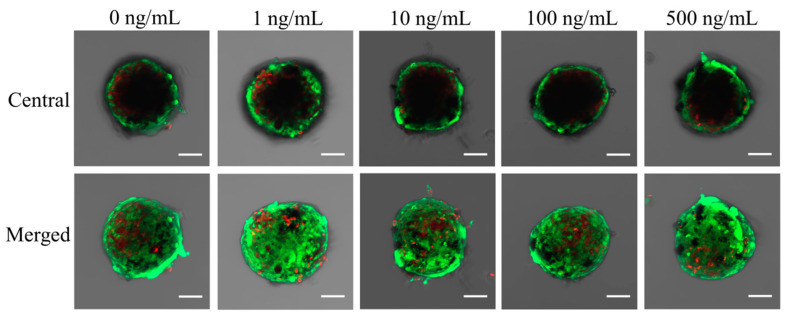
Results of central and merged images on Day 7. The length of the scale bar is 100 μm.

The results of qualitative viability of stem cells are shown in Figure 4, Figure 5 and Figure 6. Most of the stem cells in the spheroids produced high-intensity green fluorescence on Day 1 (Figure 4). A longer incubation time did not yield an observable difference in the intensity of fluorescence (Figure 6).

**Figure 7:**
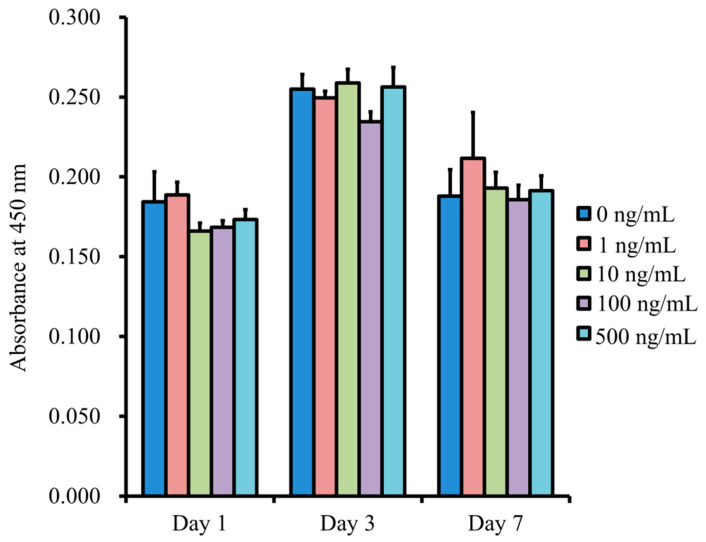
Cell viability using the Cell Counting Kit-8 on Days 1, 3, and 7.

The quantitative values for cell viability are shown in Figure 7. The absorbance values at 450 nm for NELL-1 at 0, 1, 10, 100, and 500 ng/mL concentrations were 0.184 ± 0.019, 0.189 ± 0.008, 0.166 ± 0.005, 0.168 ± 0.004, and 0.173 ± 0.006, respectively (*p* > 0.05). There were general increases in the values on Day 3, but no significant differences among 0, 1, 10, 100, and 500 ng/mL concentrations were observed (*p* > 0.05). Moreover, no differences between the groups were noted on Day 7 (*p* > 0.05).

### 3.2. Levels of Alkaline Phosphatase Activity and Anthraquinone Dye Assay

**Figure 8:**
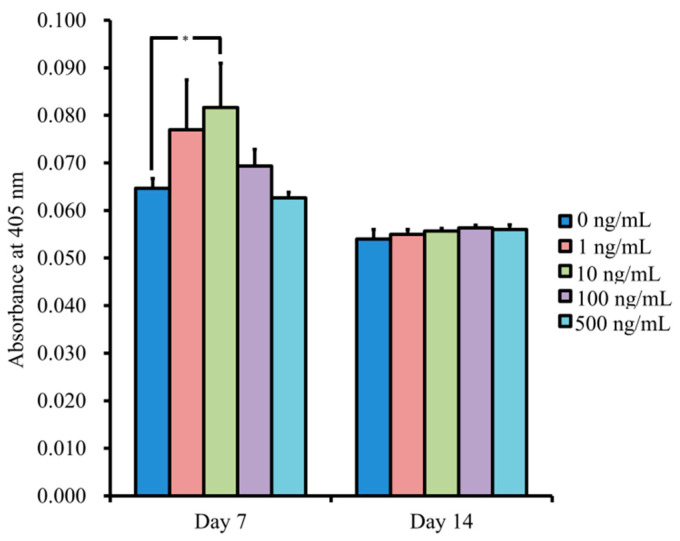
The results for alkaline phosphatase activity. * *p* < 0.05 vs. unloaded control on Day 7.

The results for NELL-1 at 0, 1, 10, 100, and 500 ng/mL concentrations on Day 7 were 0.065 ± 0.002, 0.077 ± 0.010, 0.082 ± 0.009, 0.069 ± 0.004, and 0.063 ± 0.001, respectively (Figure 8). The 10 ng/mL group showed significantly higher values when compared with the unloaded control (*p* < 0.05).

**Figure 9:**
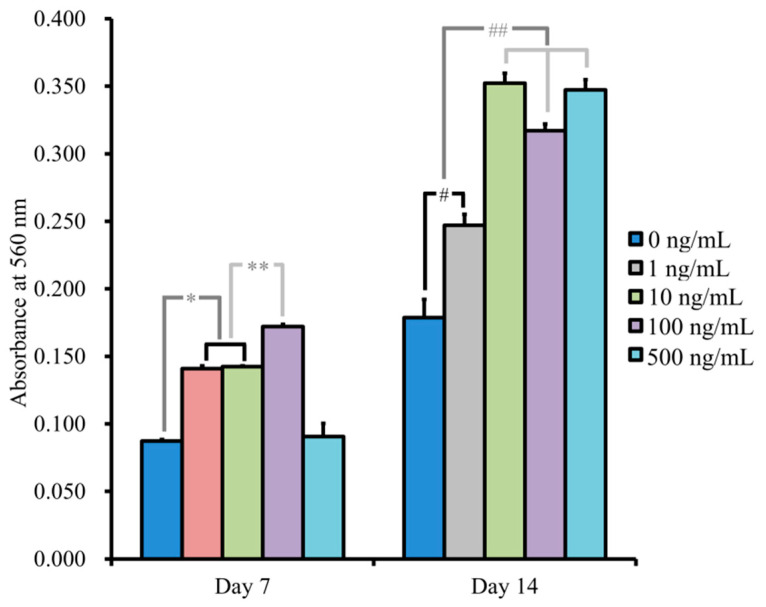
Quantification of anthraquinone dye staining. * *p* < 0.05 vs. 0 ng/mL group on Day 7. ** *p* < 0.05 vs. 1 and 10 ng/mL groups on Day 7. # *p* < 0.05 vs. 0 ng/mL group on Day 14. ## *p* < 0.05 vs. 1 ng/mL group on Day 14.

Figure 9 shows the quantitative results for anthraquinone dye staining. There were significantly higher values for NELL-1 at 1, 10, and 100 ng/mL on Day 7, with the highest value at 100 ng/mL compared with the unloaded control group (*p* < 0.05). The absorbance values at 560 nm on Day 14 for NELL-1 at 0, 1, 10, 100, and 500 ng/mL concentrations were 0.179 ± 0.014, 0.247 ± 0.008, 0.352 ± 0.007, 0.317 ± 0.005, and 0.347 ± 0.008, respectively. A significant increase was seen with addition of NELL-1, and the highest value was seen at 10 ng/mL (*p* < 0.05).

### 3.3. Evaluation of RUNX2, BSP, OCN, COL1A1 mRNA by qPCR

**Figure 10:**
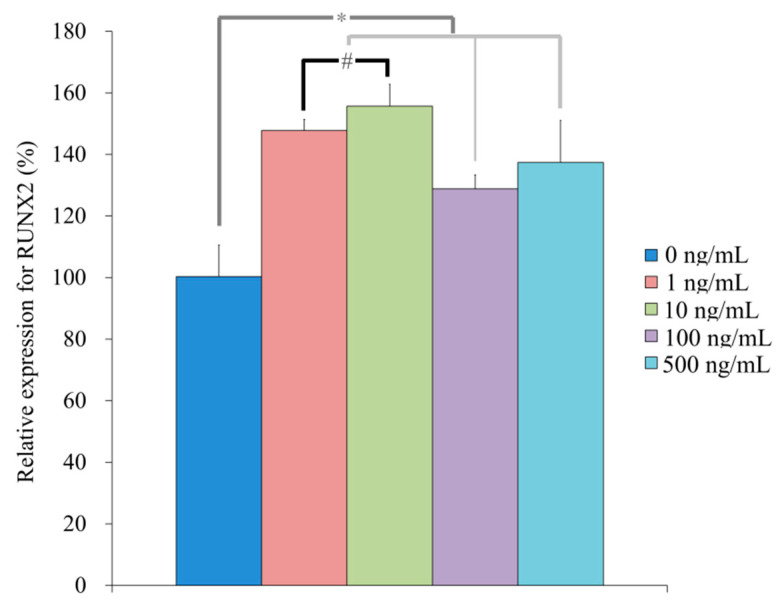
Quantification of expression of RUNX2 mRNA on Day 7. * *p* < 0.05 vs. NELL-1 at 0 ng/mL. # *p* < 0.05 vs. 10 ng/mL group.

**Figure 11:**
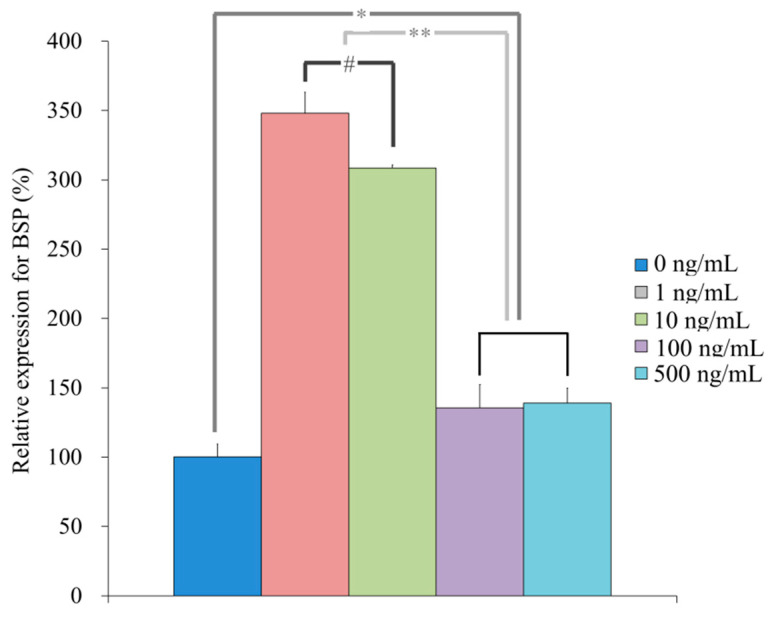
Quantification of expression of BSP mRNA on Day 7. * *p* < 0.05 vs. 0 ng/mL group. ** *p* < 0.05 vs. 1 and 10 ng/mL groups. # *p* < 0.05 vs. 1 ng/mL group.

**Figure 12:**
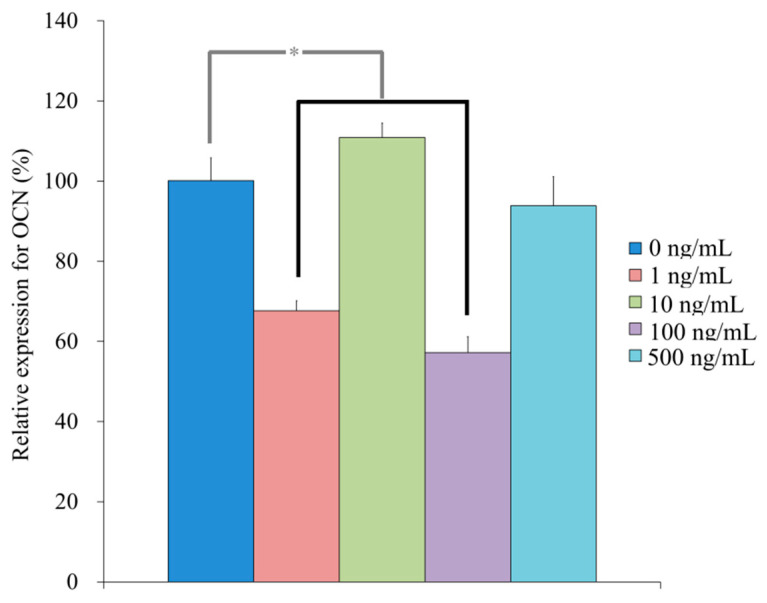
Quantification of expression of OCN mRNA on Day 7. * *p* < 0.05 vs. 0 ng/mL group.

**Figure 13:**
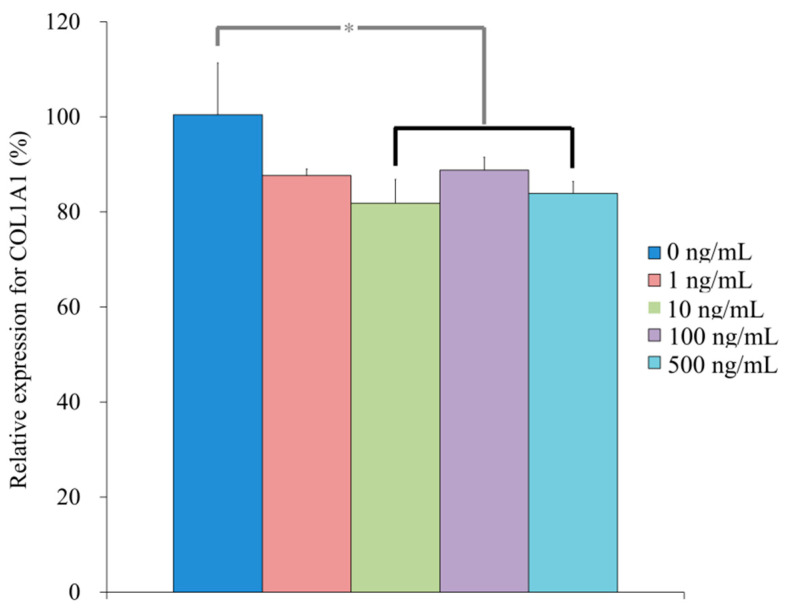
Quantification of expression of COL1A1 mRNA on Day 7. * *p* < 0.05 vs. 0 ng/mL group.

qPCR revealed that the mRNA levels of *RUNX2* were 100.4 ± 10.2, 147.7 ± 3.7, 155.7 ± 7.0, 128.8 ± 4.5, 137.4 ± 13.6 for NELL-1 at 0, 1, 10, 100, and 500 ng/mL, respectively, on Day 7 (*p* < 0.05) (Figure 10). qPCR revealed that the mRNA levels of *BSP* were 100.3 ± 9.3, 348.1 ± 15.1, 308.5 ± 2.2, 135.5 ± 16.9, and 139.0 ± 10.9 for NELL-1 at 0, 1, 10, 100, and 500 ng/mL, respectively, on Day 7 (*p* < 0.05) (Figure 11). qPCR revealed that the mRNA levels of *OCN* were 100.1 ± 5.7, 67.6 ± 2.5, 110.9 ± 3.6, 57.2 ± 4.0, and 93.8 ± 7.2 for NELL-1 at 0, 1, 10, 100, and 500 ng/mL, respectively, on Day 7 (*p* < 0.05) (Figure 12). qPCR revealed that the mRNA levels of *COL1A1* were 100.4 ± 10.9, 87.6 ± 1.4, 81.9 ± 5.0, 88.8 ± 2.7, and 83.9 ± 2.5 for NELL-1 at 0, 1, 10, 100, and 500 ng/mL, respectively, on Day 7 (*p* < 0.05) (Figure 13).

## 4. Discussion

This study reported the effects of NELL-1 on cell viability and osteogenesis using cell spheroids composed of stem cells. The application of NELL-1 increased osteogenic differentiation, which was confirmed by alkaline phosphatase activity and anthraquinone dye staining, while maintaining cell viability.

NELL-1 can be widely applied in oral and maxillofacial regions due to its effects on bone and cartilage [24]. NELL-1 enhanced osteogenic differentiation of osteoblast-like cells cultured on the surface of titanium [25]. NELL-1 groups exhibited higher bone mineral density in corticotomy-assisted tooth movement and osteogenesis using a rat model when compared with the NELL-1 untreated group [26]. In this report, NELL-1 enhanced osteogenic differentiation of stem cell spheroids. Previous reports showed that alkaline phosphatase activity was enhanced by NELL-1, with the highest value at 100 ng/mL and highest osteocalcin expression at 100 ng/mL on Day 18 [25]. In this report, the highest value was observed at 10 ng/mL for alkaline phosphatase activity on Day 7, and the results of anthraquinone dye staining showed the highest value at 10 ng/mL compared on Day 14. ALP activity is reported to be an early marker of osteogenic differentiation [27,28]. It was shown that the maximal ALP activity of stem cells was obtained on Day 7, which is similar to the present results [29].

The effects of concentration of NELL-1 have been evaluated in previous studies [15,25]. A mouse osteoblast cell line (MC3T3-E1) was treated with 1, 10, 100, and 500 ng/mL of NELL-1 [25]. A mouse bone marrow-derived stroma cell line (ST2 cell line) was treated with NELL-1 at 10, 100, and 500 ng/mL [15]. NELL-1 was applied in four dosages of 1, 10, 50, and 100 ng/mL for stem cells derived from adipose tissue [30]. High concentrations of 5 and 10 μg/mL were used for bone marrow stem cells from pigs [31]. Similarly, high concentrations ranging from 0.5 μg/mL to 50 μg/mL were used for various cells including ST2, MC3T3, C3H10T1/2, M2-10B4, and ATDC5 cells [32]. For animal models, NELL-1 was applied at 700 ng/mL with a total of 3.5 ng for pulp tissue application in non-carious upper first molars from Wistar rats [7]. Recombinant mouse NELL-1 was applied at a 1.25 mg/kg concentration by a single intravenous injection from the lateral tail [12]. For corticotomy-assisted tooth movement, NELL-1 was applied at 100 or 300 µg/mL concentration [26]. Discrepancies in optimal concentrations in osteogenic differentiations may be due to variations in culture time, culture conditions, and type of model used in each study [33].

*RUNX2* is known to be a molecular biomarker for osteoblastic differentiation [21,34]. *RUNX2* is capable of inducing the synthesis and expression of *BGLAP* and *BGLAP* is considered to be a specific marker of mature osteoblasts [35]. Collagen I was known to be an osteogenic marker osteogenic supplements led to activation of collagen I expression [36]. We found that NELL-1 treatment upregulated RunX2 and BSP and *OCN* but not *COL11A* in cell spheroids on Day 7. While this shows that NELL-1 is important for the structural changes required for cell spheroid development, the decrease in *COL1A* suggests that NELL-1 is not involved in the formation of the extracellular matrix that is formed later in bone development. However, this study contains some limitations. The tissue used in this study was obtained from an individual in old age and this could influence the results [37]. The live/dead assay may detect the cells located on the surface of the spheroids [38].

Several studies have explored the underlying mechanisms modulated by NELL-1 [5,25,39]. NELL-1 is involved in upregulating runt-related transcription factor 2, a key transcription factor associated with osteoblast differentiation [20,40]. NELL-1 is activated through the mitogen-activated protein kinase-extracellular signal-regulated kinase signaling pathway in pre-osteoblasts on titanium surfaces [25]. NELL-1 is reported to bind to integrin β1, leading to the induction of the Wnt/β-catenin signaling pathway [5]. NELL-1 produced comparable osteogenic potency with bone morphogenic protein-2, which is approved by the Food and Drug Administration [24]. However, NELL-1 is shown to enhance bone defect healing through recruitment of endogenous cells and induction of vascularization, which may differ from the mechanism of bone morphogenetic protein-2 [15]. Moreover, NELL-1 has the additional advantage of not having the off-target effects commonly seen with bone morphogenetic protein-2, leading to wider application for small and large animal models [41].

This research suggests combined use of NELL-1 and stem cell spheroids. In a previous report, a synergistic effect was observed from NELL-1, combined with adipose-derived stem cells on increasing bone formation in osteogenesis imperfecta treatment [12]. Combining NELL-1 with a smoothened agonist enhanced bone healing, with a significant increase in both bone volume and bone mineral density [42]. Modification of NELL-1 by polyethylene glycol can result in enhanced pharmacokinetics for systemic therapy [43]. Long-term expression of NELL-1 can be achieved by viral mediation, which can eliminate or minimize multiple administrations or dose escalation [12].

The results showed that NELL-1 produced increased osteogenic differentiation without affecting cell viability with increased mRNA expression levels of RUNX2 and BSP. Based on these findings, NELL-1 can be applied for increased osteogenic differentiation of stem cell spheroids.

## Data Availability

All data analyzed during this study are included in this published article.
